# Circular RNA hsa_circ_0075323 promotes glioblastoma cells proliferation and invasion via regulation of autophagy

**DOI:** 10.1186/s13008-023-00084-9

**Published:** 2023-01-17

**Authors:** Wenrui Zhang, Zhonggang Shi, Shouren Chen, Shaoshan Shen, Songjie Tu, Jian Yang, Yongming Qiu, Yingying Lin, Xuejun Dai

**Affiliations:** 1 Brain Injury Center, Department of Neurosurgery, Ren Ji Hospital, School of Medicine, Shanghai Jiao Tong University, Room 1626, Building 17, 1630 Dongfang Road, Pudong Distric, Shanghai, 200127 People’s Republic of China; 2 Department of Neurosurgery, Zhangzhou Affiliated Hospital of Fujian Medical University, NO. 59th West Shengli Road, Zhangzhou, 363000 China

**Keywords:** hsa_circ_0075323, Glioblastoma, Proliferation, Invasion, Autophagy

## Abstract

**Background:**

Protein p62 (sequestosome 1) encoded by gene SQSTM1 plays a vital role in mediating protectively selective autophagy in tumor cells under stressed conditions. CircSQSTM1 (hsa_circ_0075323) is a circular transcript generated from gene SQSTM1 (chr5:179260586–179260782) by back-splicing. However, the potential role of hsa_hsa_circ_0075323 in glioblastoma (GBM) remains unclear. Here, we aimed to explore the biological function of hsa_circ_0075323 in GBM and its relationship with autophagy regulation.

**Results:**

Hsa_circ_0075323 is highly expressed in GBM cells and mainly locates in the cytoplasm. Inhibition of hsa_circ_0075323 in U87-MG and T98G cells attenuated proliferation and invasion ability significantly, while upregulation of has_ circ_0075323 enhanced proliferation and migration of U251-MG and A172 cells. Mechanistically, depletion of hsa_circ_0075323 in GBM cells resulted in impaired autophagy, as indicated by increased expression of p62 and decreased expression of LC3B.

**Conclusions:**

Hsa_circ_0075323 regulates p62-mediated autophagy pathway to promote GBM progression and may serve as a prognostic biomarker potentially.

## Introduction

Every year, about 100,000 people are diagnosed with diffuse glioma worldwide [1]. Although diffuse glioma accounts for less than 1% of newly diagnosed cancer cases globally, it remains a severe threaten to human health because of the high mortality and morbidity [2]. Glioblastoma (GBM), regarded as the most aggressive type in diffuse glioma, accounts for 70 ~ 75% in total cases approximately [3]. According to U.S. reports, GBM accounts for 14.6% of primary brain and other central nervous system (CNS) tumors and 48.3% of malignant ones, with a five-year relative survival rate below 10% [4]. As a WHO grade IV astrocytoma, GBM exhibits higher degree of malignancy with greatly infiltrating into surrounding brain tissues, so it is difficult to fully remove tumor tissue through surgical operations considering its ambiguous boundaries [5]. Over the past two decades, recombination of temozolomide and radiotherapy has improved average two-year survival rate of GBM to 27%. However, the existence of glioma stem-like cells (GSCs), which associate with radio-resistance and recurrence post radiotherapy, lead to poor clinical outcomes eventually [6–9]. Recent years, innovative immune and targeted therapies have triggered interests of more and more researchers in this field, and identified potential molecular targets for GBM treatment is imperative [10, 11].

Autophagy receptor p62 is encoded by gene SQSTM1 on chromosome 5 and participates in selective autophagy upon cellular stress [12]. In epithelial cells, enhanced expression of protein p62 is sufficient to induce malignant transformation and initialize tumor [13]. But in stromal cells of the tumor microenvironment, p62 mainly functions as a tumor suppressor by attenuating inflammation and fibrosis [14]. Generally, homeostatic maintenance of p62 expression levels in both tumor and stroma may decide the final outcomes of tumor initiation and progression through complex regulatory network involving mTORC1/Myc, NRF2 and NF-κB signaling pathways [15, 16]. Current studies mostly focus on autophagy correlated or uncorrelated functions of p62 when referred to gene SQSTM1, however, few researchers have paid attention to the function of its transcripts, especially circular RNA [17].

Circular RNA (circRNA) is a novel class of functional non-coding RNA recognized ubiquitous in eukaryotes [18]. Being different from their linear counterparts, circRNA contain a covalently closed loop structure through a typical 3’ to 5’ phosphodiester bond [19]. Without free terminus, circRNA are highly stable and found to be more abundant than mRNA in thousands of human genes [20, 21]. In particular, circRNA show great enrichment and stableness in extracellular fluid, which makes them ideal candidates as cancer biomarkers [22]. In mammalian brains, circRNA are highly conserved and dynamically expressed, hinting at their important biological functions in mediating brain development and disease occurrence [23]. So far, multiple physiological functions of circRNA have been identified involving interactions with microRNA (miRNA), protein and DNA [24]. Briefly, circRNA serve as regulators of miRNA functions by selective sponging [25]. Besides, circRNA can bind to RNA-binding proteins (RBPs) and act as sponges, decoys or scaffolds functionally [26]. In human brains, altered expression of circRNA is believed to mediate severe disorders including tumorigenesis and neurodegenerative disorders like Alzheimer’s disease [27, 28]. However, how circRNA regulate gene function of SQSTM1 and whether the underlying mechanisms play a role in GBM onset and progression remain elusive.

Here, we have identified a circular RNA generated at the SQSTM1 gene locus, termed hsa_circ_0075323. Hsa_circ_0075323 is widely expressed in A549, AG04450, BJ, HELAS3, HepG2, HUVEC, NHEK and SKNSHRA, but also specially expressed in human neuroblastoma cell [29]. Our further study found that the expression of hsa_circ_0075323 is significantly higher in GBM cells compared with normal astrocytes. Mechanistically, hsa_circ_0075323 promotes tumor proliferation and invasion through regulating autophagy pathway in association with protein p62. Taken together, our findings revealed a fresh function of hsa_circ_0075323 in GBM progression partly by influencing p62-mediated autophagy.

## Materials and methods

### Cell culture

Normal human astrocytes (HEB) and human glioblastoma cell lines (U87-MG, U251-MG, A172 and T98G) were purchased from ATCC (American Type Culture Collection). Cells were cultured in Dulbecco’s Modified Eagle’s Medium (DMEM) (Gibco, Germany) supplemented with 10% fetal bovine serum (Gibco), 100 U/ml penicillin and 100 μg/ml streptomycin (Gibco) in a humidified atmosphere at 37 ℃ containing 5% CO_2_.

### Lentivirus-mediated knockdown or overexpression of Hsa_circ_0075323

Recombinant lentivirus containing hsa_circ_0075323 coding sequence or specific siRNA sequence (si-hsa_circ_0075323, 5ʹ-GCCAGGAACAGATGGAGTC-3ʹ) or non-specific negative control oligos (si-NC) were constructed. To ectopic express hsa_circ_0075323, a basic sequence flanked by XhoI and Agel was synthesized. A small spacer sequence containing two restriction enzyme sites, HindIII and SalI, was added for the insertion of circRNA fragment to the plasmid vector. Lentivirus package and purification were handed over to Hanyin Co. (Shanghai, China) and the virus titer reached over 10^9^ TU (transfection unit)/mL before use. GBM cells were resuscitated and cultured in 6 cm cell culture dishes. The cells were subcultured in 6-well plates until the density reached 80% (3–5 × 10^4^ cell/ml, 2 ml). Lentivirus infection was carried out in the presence of 8 μg/m polybrene. U87-MG and T98G cells were infected with lentivirus carrying si-hsa_circ_0075323, while A172 and U251-MG cells were infected with lentivirus carrying hsa_circ_0075323 sequence. The culture medium was replaced with selection medium containing 4 μg/ml puromycin after 72 h and then the cells were cultured for another 14 days. The puromycin-resistant cells were amplified in medium containing 2 μg/ml puromycin for seven days and then transferred to medium without puromycin.

### Quantitative real-time RCR (qRT-PCR)

Total RNA was extracted using TRIzol and reverse transcribed using Prime Script RT Master Mix (Takara, Japan). PCR was performed using PCR Master Mix (2 ×) (Thermo Fisher Scientific, Waltham, MA, USA), primer sequences for hsa_circ_0075323 were synthesized as following: forward 5ʹ-ACATCTCCCGCCAGGAACA-3ʹ; reverse 5ʹ-CCTGTAGACGGGTCCACTTC-3ʹ. To quantify the amounts of hsa_circ_0075323, real-time PCR analyses were performed using a SYBR Premix Ex TaqTM kit (Takara, Japan) with GAPDH as internal controls. Each sample was replicated three times and data were analyzed by comparing Ct values.

### RNA fluorescence in site hybridization (FISH)

Cy3-labeled human hsa_circ_0075323 probe was synthesized by Ribo Bio. and applied for FISH. A Fluorescent In Situ Hybridization Kit (C10930, RiboBio, Guangzhou, China) containing pre-hybridization buffer, hybridization buffer and 4,6-diamidino-2-phenylindole (DAPI) was used and the assay was performed according to the manufacturer’s instructions. The nuclei were stained with DAPI. 18S and U6 served as internal references while the former almost entirely localized in cytoplasm and the latter in nuclei. The images were photographed under fluorescence microscope (Leica, SP8 laser confocal microscope).

### Cell proliferation assay

Lentivirus-infected cells were seeded into a 96-well plate at a density of 2000 cells per well and the assay was performed using the Cell Counting Kit 8 (CCK-8, Dojindo) according to the manufacturer’s protocol. The optical density of each well was measured at 450 nm using a microplate spectrophotometer (Epoch 2, BioTek, USA).

### Migration assay

A Boyden chamber system (Costar Corp., Cambridge, Mass, US) was purchased for Transwell migration assay. Cells (3 × 10^4^) were seeded into each insert and incubated for 24 h. The cells remained in the top of the inserts were removed and migrating cells were fixed with 75% ethanol for 30 min followed by 0.1% crystal violet staining for 20 min. The cells migrating to the lower chamber were counted and photographed by microscope (Leica DMI 400B).

### Western blotting analysis

HEB, U87-MG, U251-MG, A172 and T98G cells were harvested and extracted with RIPA buffer containing protease inhibitors. Protein concentration was determined using a bicinchoninic acid protein assay kit (Beyotime, P0010). The immunoreactive bands were detected using an ECL kit (Pierce, PI32209). Primary antibodies targeting the following proteins were applied: LC3B (#3868, Cell signaling technology), p62 (#39749, Cell signaling technology) and actin (MA1-744, Invitrogen). HRP-conjugated goat anti-rabbit (cat. no. SA00001-2) antibodies were used as secondary antibodies (Proteintech, USA). Semi-quantitative analysis was performed by ImageJ software (NIH, version1.8.0).

### Statistical analysis

Data of this study are presented as the mean ± SD from at least 3 replicates. Student’s two-tailed unpaired *t*-test was used to determine differences between 2 groups. All statistical analyses were performed using SPSS software (Windows, v17.0.). Significance was defined as *P* < 0.05.

## Results

### Hsa_circ_0075323 was highly expressed in GBM cells

**Fig. 1:**
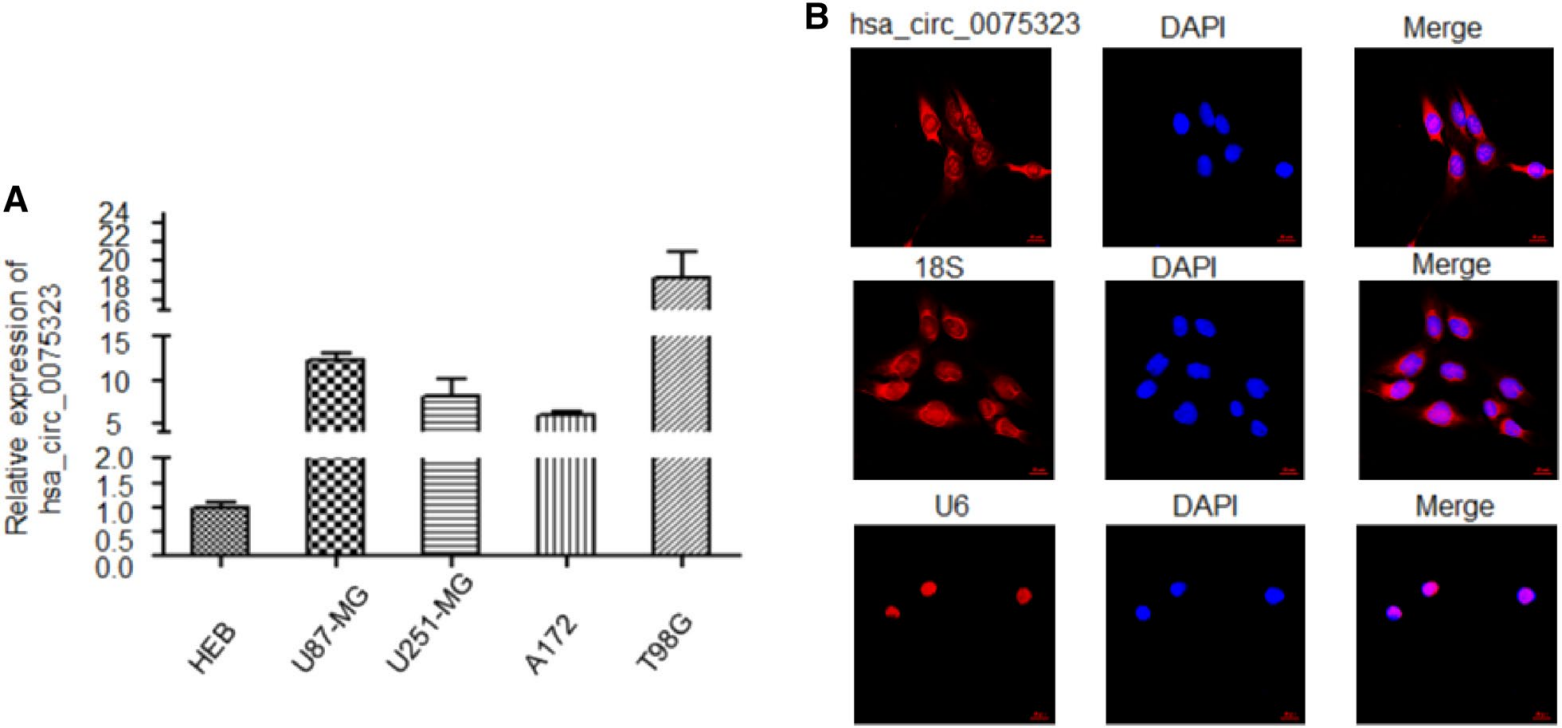
hsa_circ_0075323 expression is higher in GBM cells. **A**.Relative expression of hsa_circ_0075323 tested by qRT-PCR in HEB, U87-MG, U251-MG, A172 and T98G cells. **B** Subcellular localization of hsa_circ_0075323 identified by FISH. The nuclei were stained with DAPI, 18S and U6 served as internal references

We examined the expression levels of hsa_circ_0075323 in GBM cells (U87-MG, U251-MG, A172 and T98G) and corresponding normal human astrocytes (HEB) by qRT-PCR. As a result, expression of hsa_circ_0075323 turned out to be extraordinarily higher in GBM cells compared with that in HEB (Fig. 1A). We then divided the four GBM cell lines into high-expression group (U87-MG and T98G) and middle-expression group (A172 and U251-MG) depending on endogenous level of hsa_circ_0075323 for subsequent studies. Meanwhile, we detected the subcellular localization of hsa_circ_0075323 in GBM cells (U87-MG, U251-MG, A172 and T98G) by FISH. It was found that hsa_circ_0075323 was mainly present in the cytoplasm (Fig. 1B).

### Hsa_circ_0075323 was successfully overexpressed or knockdown in GBM cells

**Fig. 2:**
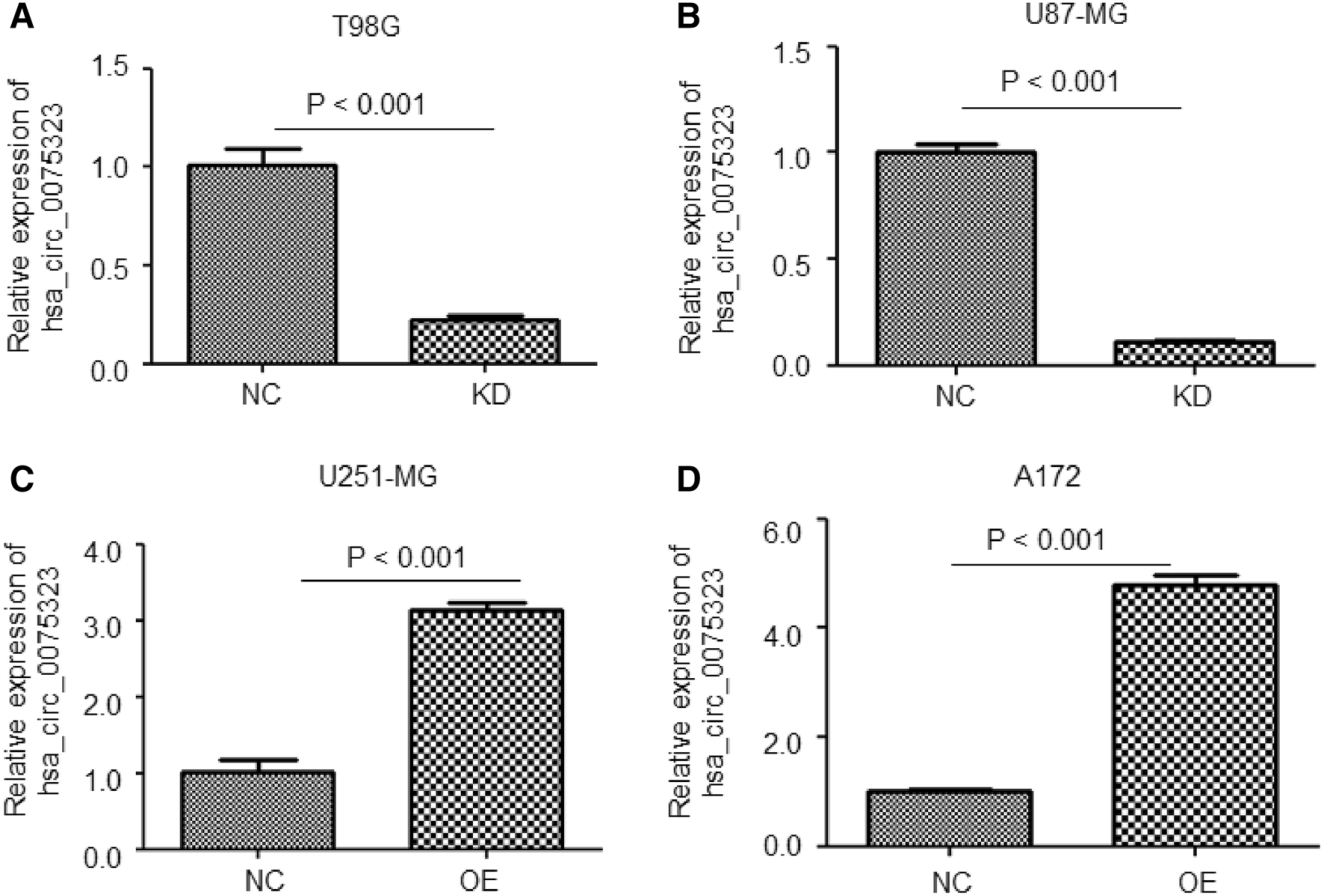
hsa_circ_0075323 was successfully overexpressed or knockdown in GBM cells. **A** and **B**, relative expression of hsa_circ_0075323 in T98G and U87-MG cells after lentivirus-mediated knockdown. **C** and **D**, relative expression of hsa_circ_0075323 in U251-MG and A172 cells after lentivirus-mediated overexpression

We selectively performed hsa_circ_0075323 knockdown in T98G and U87-MG cells by lentivirus infection. By similar method, hsa_circ_0075323 was overexpressed in U251-MG and A172 cells. Quantitative analysis showed significantly decreased expression of circ_007532 in T98G and U87-MG cells (Fig. 2A, B). As expected, hsa_circ_0075323 was significantly upregulated in U251-MG and A172 cells after treatment (Fig. 2C, D).

### Downregulation of hsa_circ_0075323 attenuates proliferation and invasion of GBM cells

**Fig. 3:**
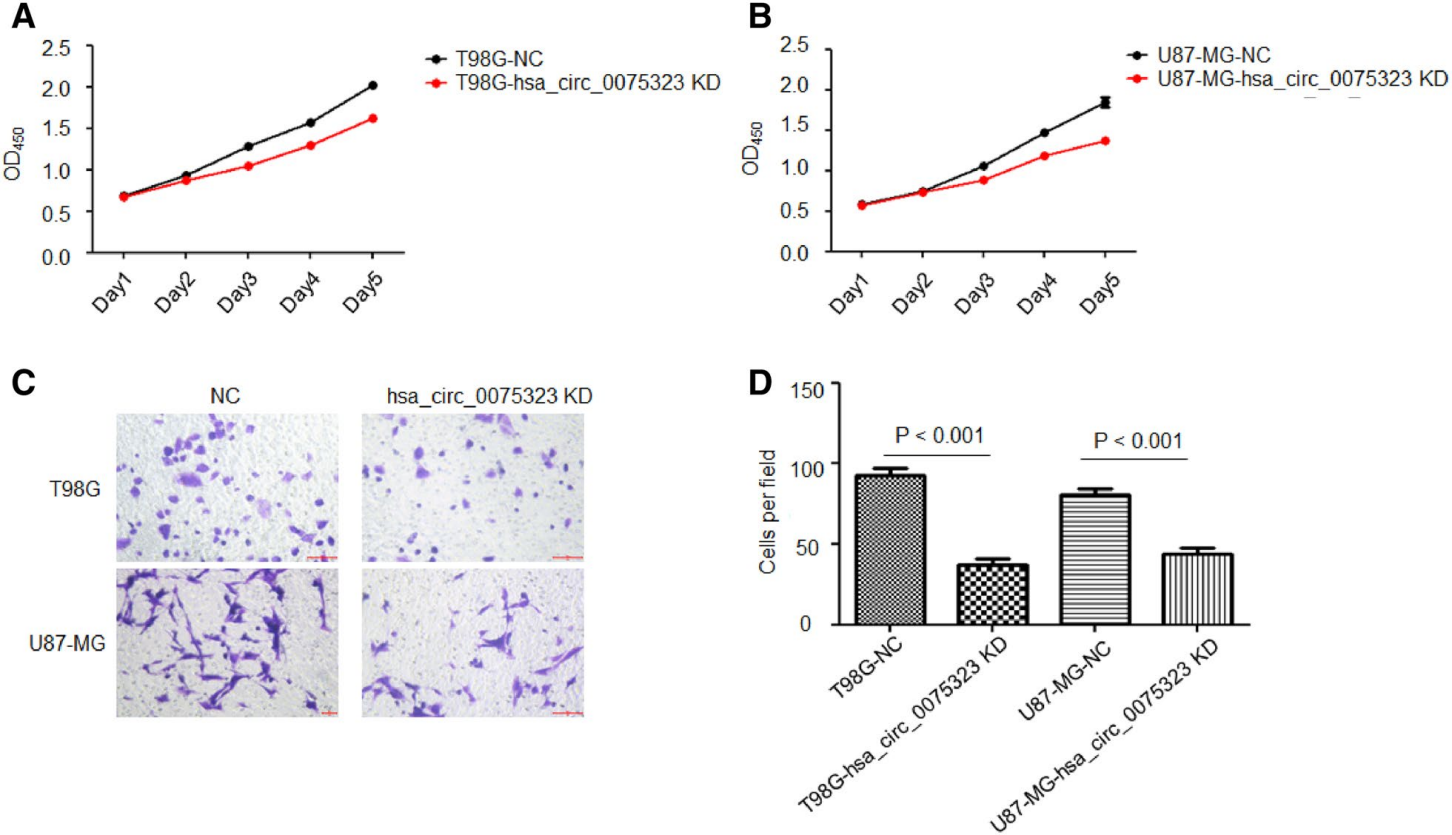
Downregulation of hsa_circ_0075323 attenuates proliferation and invasion of GBM cells. **A** and **B**, Cell proliferation was determined by CCK-8 assay in T98G and U87-MG cells transfected with si- circ_0075323 lentivirus. **C** and **D**, Cell invasion ability was determined by Transwell migration assay in T98G and U87-MG cells transfected with si- circ_0075323 lentivirus

CCK-8 assay was carried out to figure out the effect of hsa_circ_0075323 on GBM cells proliferation. It turned out that hsa_circ_0075323 inhibition decreased proliferation in both T98G and U87-MG cells (Fig. 3A, B). Beyond that, transwell migration assay demonstrated that cell invasive potential was significantly weakened by hsa_circ_0075323 knockdown in both T98G and U87-MG cells (Fig. 3C, D).

### Upregulation of hsa_circ_0075323 promoted proliferation and invasion of GBM cells

**Fig. 4:**
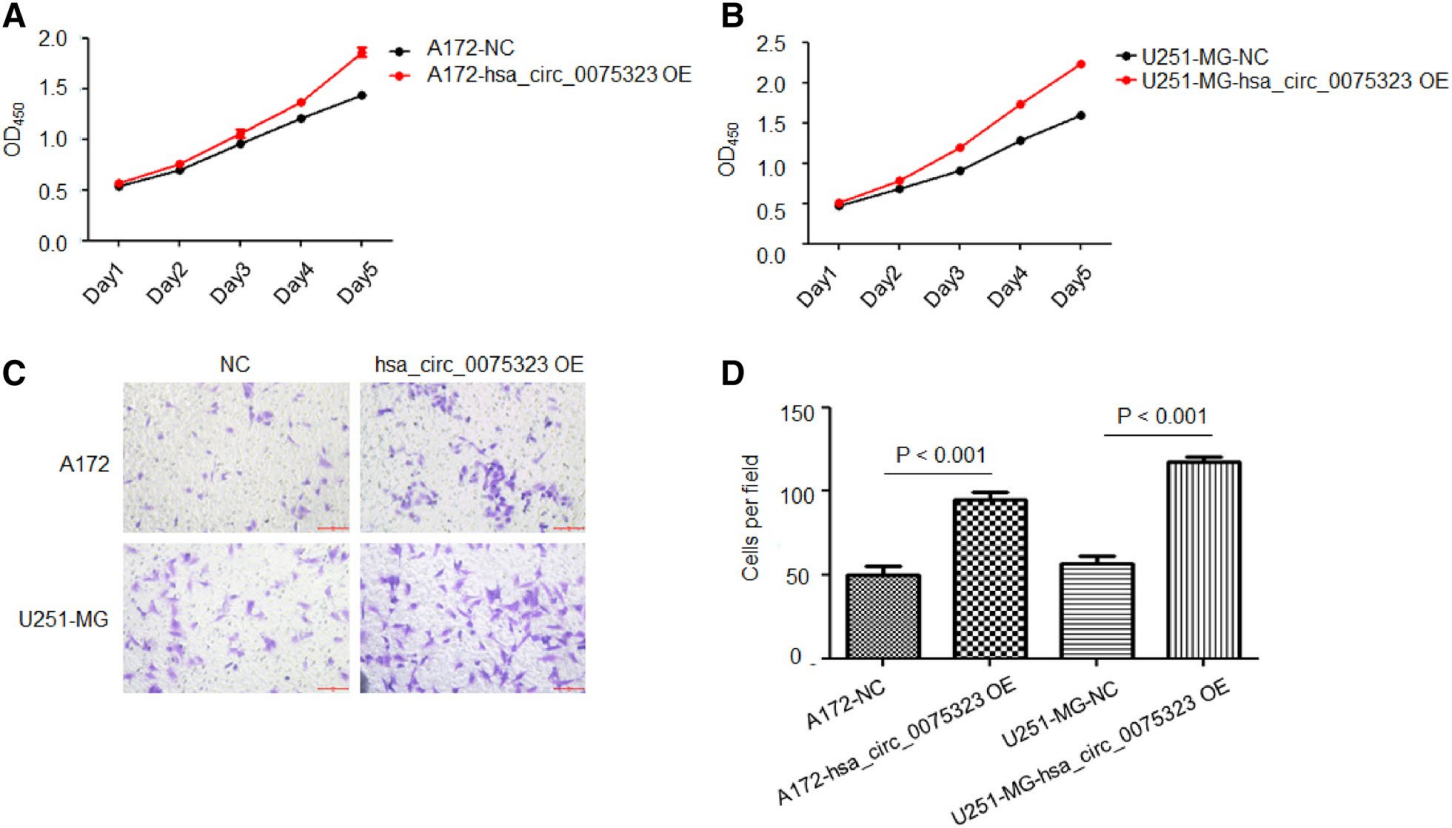
Upregulation of hsa_circ_0075323 promoted proliferation and invasion of GBM cells. **A** and **B**, Cell proliferation was determined by CCK-8 assay in A172 and U251-MG cells infected with circ_0075323 lentivirus. **C** and **D**, Cell invasion ability was determined by Transwell migration assay in A172 and U251-MG cells transfected with circ_0075323 lentivirus

On the contrary, we assessed the effect of hsa_circ_0075323 overexpression on GBM cells. The results indicated that upregulation of hsa_circ_0075323 significantly enhanced proliferation and invasion in A172 and U251-MG cells (Fig. 4A–D).

### Hsa_circ_0075323 regulated p62 expression and autophagy

**Fig. 5:**
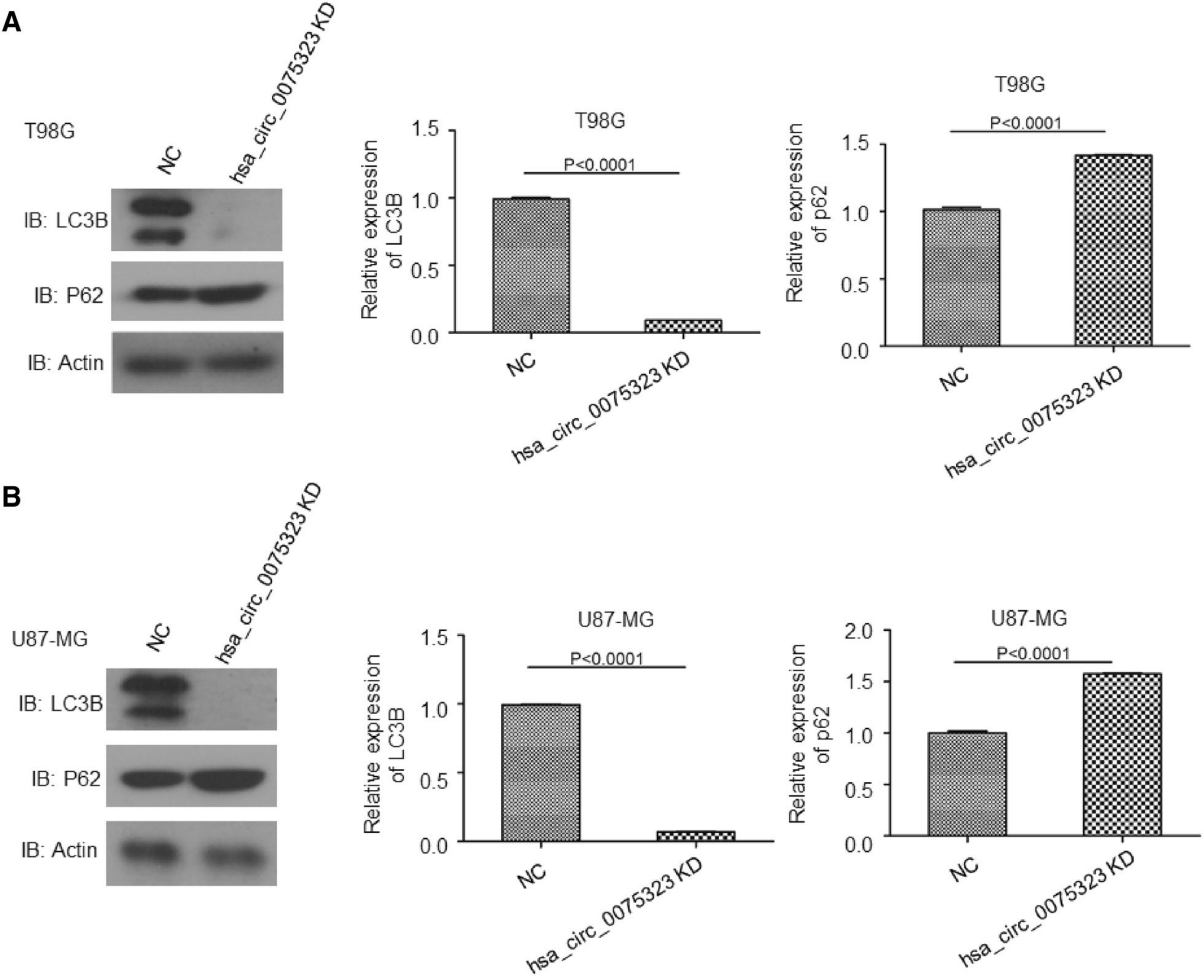
hsa_circ_0075323 regulated p62 expression and autophagy. **A** and **B**, Relative expression of protein LC3B and p62 were tested by Western Blotting in T98G and U87-MG cells infected with si-circ_0075323 lentivirus. Semi-quantitative analysis of protein levels was performed by ImageJ

Autophagy plays critical roles in sustaining GBM cells survival and proliferation, especially under stressed conditions including hypoxia, nutrient deprivation and radiation or temozolomide (TMZ) treatment [30]. LC3B is the most studied member of the mammalian ATG8 protein family [31]. Commonly, LC3B is regarded as an indicator of active autophagy, considering its indispensable role in autophagosome development and maturation [32]. Hence, we assessed whether hsa_circ_0075323 regulated autophagy activity in GBM cells. Total proteins were extracted from hsa_circ_0075323 knockdown T98G and U87-MG cells. Expressions of protein p62 and autophagy-associated protein LC3B were tested by Western Blotting. In T98G and U87-MG cells, hsa_circ_0075323 inhibition significantly limited expression of LC3B, which could hardly be detected under our experiment conditions. Conversely, p62 expression was significantly promoted after hsa_circ_0075323 depletion in these cells (Fig. 5A, B).

## Discussion

The cellular roles of circRNAs have become a focus of cancer biology. Most attention was given to its roles on protein expression from modulating transcription in the nucleus to translation in the cytoplasm. In our study, we demonstrated that hsa_circ_0075323 acts as an activator of GBM progression as shown in gain-of- and loss-of-functional assays. In additional, has_circ_0075323 regulates autophagy signaling pathway by modulating the protein expression of SQSTM1 as shown in molecular assays. Taken together, we are the first to identify tumor-promoting effect of hsa_circ_0075323 in GBM and the first to reveal underlying mechanisms in association with autophagy regulation.

Several emerging experimental approaches help researchers update biological functions of circular RNA. Not only circular RNA could sponge for miRNA or proteins, form functional circRNP complexes, it could interact with mRNAs to affect their expression and even outcompete linear mRNAs for protein binding in the cytosol [33]. Approximately 20% protein-coding genes in brains could generate circRNA, and many host genes which produce circRNA are exclusively expressed in brains, which means their roles in mammalian brains is exceptional and complex [23]. In our studies, we identify has_circ_0075323 mainly located in the cytoplasm. Meanwhile, the depletion of hsa_circ_0075323 resulted in enhanced expression of protein p62. This kind of autoregulating mechanism is not rare across human genome. It has been discovered that circMBL derived from gene Musclebind (MBL) could specifically bound with protein MBL as a sponger. In turn, protein MBL could promote production of circMBL whose upregulation would mask the effects of protein MBL [34]. Whether a similar inter-regulatory feedback loop exists between protein p62 and hsa_circ_0075323 needs further investigation.

**Fig. 6:**
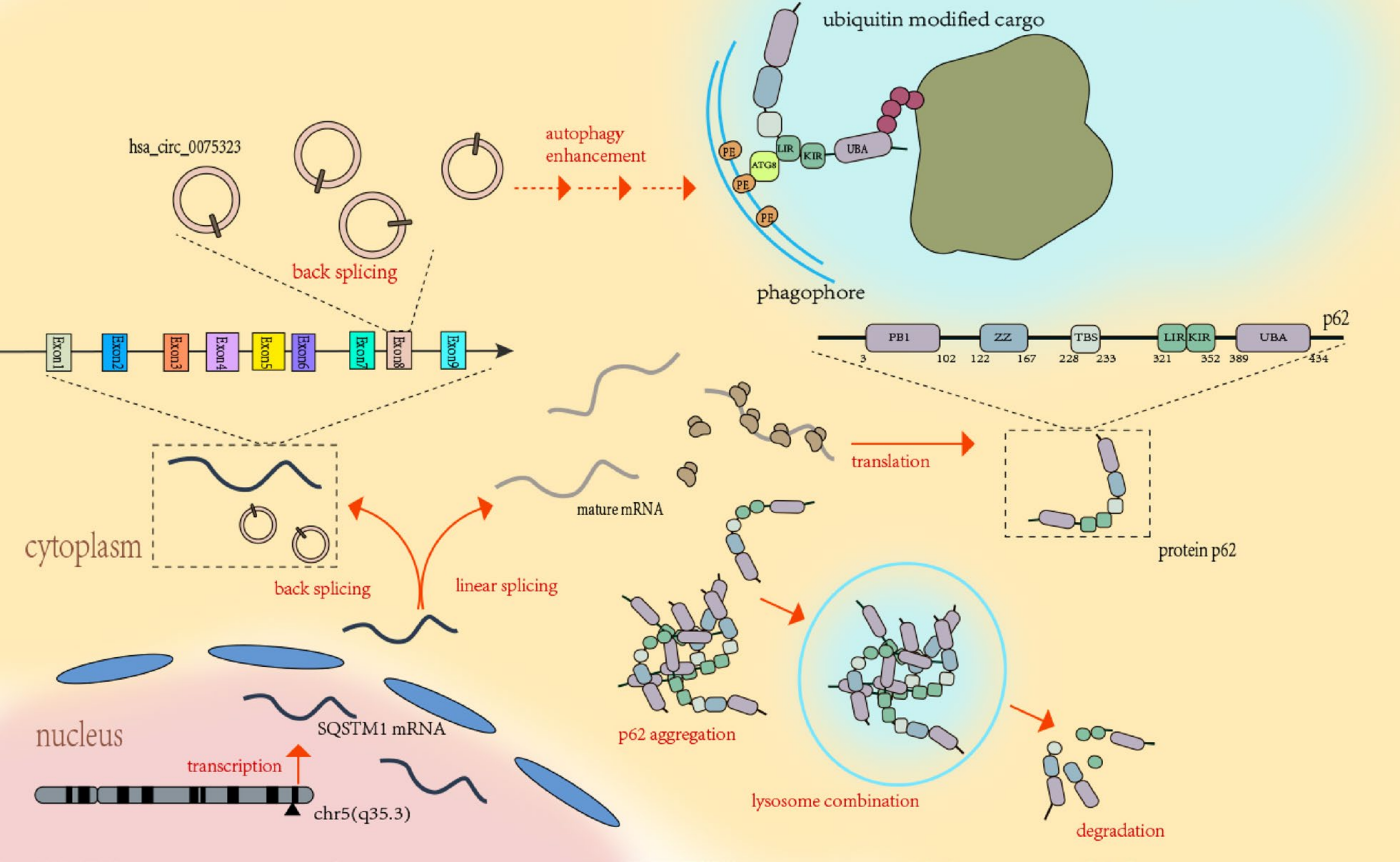
A sketch map of hsa_circ_0075323 relevant molecular events in regards of autophagy regulation

Both LC3B and p62 are components of core autophagy machinery which is essential for autophagosome formation. There are three main types of autophagy named macroautophagy, microautophagy and chaperone-mediated autophagy (CMA) in eukaryocytes [35]. The macroautophagy (often referred to as autophagy) is a stepwise process mediated by autophagy receptors to selectively degrade large intracellular cargoes like protein aggregates and damaged organelles [36]. Upon autophagy initiation, p62 interacts with ubiquitin modified cargoes through its ubiquitin-associated (UBA) domain on C-terminal. On the other side, LC3-interacting region (LIR) motif of p62 interacts with ATG8 family proteins including LC3B. The lipidation of ATG8 family proteins to phosphatidylethanolamine (PE) on phagophore inner membrane anchors selected cargoes to a growing phagophore. However, in normal conditions when autophagy substrates are insufficient, intracellular p62 is “supersaturated” and tends to degrade by selective autophagy [37, 38]. We summarized hsa_circ_0075323 relevant molecular events in regards of autophagy regulation by a sketch map (Fig. 6). As shown in our study, expression level of p62 and LC3B correlated conversely in response to hsa_circ_0075323 inhibition in GBM cells. One assumption is that excess p62 is more apt to polymerize and degrade. The imbalance between protein levels of p62 and LC3B is unfavorable for phagosome formation and therefore results in inhibited autophagy. Thus, we suppose that hsa_circ_0075323 is a vital regulator for maintaining dynamic equilibrium of intracellular p62 content, which influences outcomes of autophagy eventually. As protective autophagy is prevalent in radio-resistant or (and) chemo-resistant GBM cells [39], hsa_circ_0075323 may serve as a prognostic biomarker or a potential treatment target for radio-resistant or chemo-resistant GBM hopefully.

## Limitations and prospects

In our study, we identified a pro-cancer role for hsa_circ_0075323 in GBM cells, suggesting a potential prognostic biomarker role, but this speculation still lacks solid data support. RNA-seq of GBM clinical samples and GBM cell lines is needed to prepare, which is helpful to verify the conclusion of in vitro cell experiments in this study. Correspondingly, the correlation between the abundance of hsa_circ_0075323 in examined tissues and patient overall survival should also be studied. The outcome of these two key issues will deepen our understanding of hsa_circ_0075323 in GBM and its translation.

The fact that circular RNAs differ from linear RNAs in conformation, stability and immunogenicity has prompted many attempts to develop circRNA-based technologies. These include the use of circRNA as non-coding adaptors to interfere with mRNA or proteins of core genes in intracellular processes, be a booster or inhibitor of innate immune responses, serve as templates for antisense RNA and extended translation and classically, serve as pathological targets and biomarkers. In-depth exploration the roles of hsa_circ_0075323 in autograph through in vivo experiments, combined with high-throughput sequencing, will help to uncover the application of hsa_circ_0075323 in GBM.

## Data Availability

The data used in the present study are available from the corresponding author upon reasonable requests.

## Abbreviations

GBM: Glioblastoma
CNS: Central nervous system
GSCs: Glioma stem-like cells
TMZ: Temozolomide
PE: Phosphatidylethanolamine
CMA: Chaperone-mediated autophagy
